# Chronic, not acute, skin-specific inflammation promotes thrombosis in psoriasis murine models

**DOI:** 10.1186/s12967-015-0738-z

**Published:** 2015-12-16

**Authors:** Jackelyn B. Golden, Yunmei Wang, Yi Fritz, Doina Diaconu, Xiufen Zhang, Sara M. Debanne, Daniel I. Simon, Thomas S. McCormick, Nicole L. Ward

**Affiliations:** Department of Dermatology, Case Western Reserve University, 10900 Euclid Ave, Cleveland, OH 44106 USA; Department of Pathology, Case Western Reserve University, Cleveland, OH USA; Harrington Heart and Vascular Institute, University Hospitals Case Medical Center, Case Western Reserve University School of Medicine, Cleveland, OH USA; Department of Epidemiology and Biostatistics, Case Western Reserve University, Cleveland, OH USA; The Murdough Family Center for Psoriasis, Case Western Reserve University, Cleveland, OH USA

**Keywords:** Psoriasis, Mouse model, Cardiovascular disease, Chronic inflammation, IL-17C, Imiquimod/Aldara, Thrombosis, Monocytosis, Skin, Neutrophils

## Abstract

**Background:**

Psoriasis patients exhibit an increased risk of atherothrombotic events, including myocardial infarction and stroke. Clinical evidence suggests that psoriasis patients with early onset and more severe disease have the highest risk for these co-morbidities, perhaps due to the extent of body surface involvement, subsequent levels of systemic inflammation, or chronicity of disease. We sought to determine whether acute or chronic skin-specific inflammation was sufficient to promote thrombosis.

**Methods:**

We used two experimental mouse models of skin-specific inflammation generated in either an acute (topical Aldara application onto wild-type C57Bl/6 mice for 5 days) or chronic (a genetically engineered K5-IL-17C mouse model of psoriasiform skin inflammation) manner. Arterial thrombosis was induced using carotid artery photochemical injury (Rose Bengal-green light laser) and carotid artery diameters were measured post-clot formation. We also examined measures of clot formation including prothrombin (PT) and activated partial thromboplastin time (aPTT). Skin inflammation was examined histologically and we profiled plasma-derived lipids. The number of skin-draining lymph-node (SDLN) and splenic derived CD11b^+^Ly6C^high^ pro-inflammatory monocytes and CD11b^+^Ly6G^+^ neutrophils was quantified using multi-color flow cytometry.

**Results:**

Mice treated with topical Aldara for 5 days had similar carotid artery thrombotic occlusion times to mice treated with vehicle cream (32.2 ± 3.0 vs. 31.4 ± 2.5 min, p = 0.97); in contrast, K5-IL-17C mice had accelerated occlusion times compared to littermate controls (15.7 ± 2.1 vs. 26.5 ± 3.5 min, p < 0.01) while carotid artery diameters were similar between all mice. Acanthosis, a surrogate measure of inflammation, was increased in both Aldara-treated and K5-IL-17C mice compared to their respective controls. Monocytosis, defined as elevated SDLN and/or splenic CD11b^+^Ly6C^high^ cells, was significantly increased in both Aldara-treated (SDLN: 3.8-fold, p = 0.02; spleen: 2.0-fold, p < 0.01) and K5-IL-17C (SDLN: 3.4-fold, p = 0.02; spleen: 3.5-fold, p < 0.01) animals compared to controls while neutrophilia, defined as elevated SDLN and/or splenic CD11b^+^Ly6G^+^ cells, was significantly increased in only the chronic K5-IL-17C model (SDLN: 11.6-fold, p = 0.02; spleen: 11.3-fold, p < 0.01). Plasma-derived lipid levels, PT and aPTT times showed no difference between the Aldara-treated mice or the K5-IL-17C mice and their respective controls.

**Conclusions:**

Chronic, but not acute, skin-specific inflammation was associated with faster arterial thrombotic occlusion. Increased numbers of splenic and SDLN monocytes were observed in both acute and chronic skin-specific inflammation, however, increased splenic and SDLN neutrophils were observed only in the chronic skin-specific inflammation model. Understanding the cellular response to skin-specific inflammation may provide insights into the cellular participants mediating the pathophysiology of major adverse cardiovascular events associated with psoriasis.

**Electronic supplementary material:**

The online version of this article (doi:10.1186/s12967-015-0738-z) contains supplementary material, which is available to authorized users.

## Background

Psoriasis vulgaris is a chronic inflammatory skin disease that affects ~2 % of Americans and is characterized by red, scaly, well-demarcated plaques containing activated immune cells [1]. The disease is associated with numerous co-morbidities including psoriatic arthritis [2], diabetes [3], kidney disease [4], and metabolic syndrome [5]. Psoriasis patients also experience increased depression and societal stigmatization [6]. Importantly, individuals with psoriasis have an increased risk of developing and dying of cardiovascular disease (CVD) [7, 8]. Likewise, patients with severe psoriasis have an increased risk of experiencing an adverse cardiovascular event, such as stroke or myocardial infarction (MI) [9], and this occurs independent of other CVD risk factors including age, gender, smoking, diabetes, hypertension, and hyperlipidemia [8]. Causality between psoriasis and CVD is challenging to explore; however, many commonalities exist at the cellular and molecular levels between the two diseases [10, 11] and treating psoriasis patients with systemic anti-inflammatory drugs may improve associated CVD outcomes [12].

Recently, we generated and reported a murine model of psoriasis driven by keratinocyte-specific overexpression of Interleukin (IL)-17C, the K5-IL-17C mouse [13]. IL-17C is an IL-17 family member believed to signal through the receptors IL-17RE/RA [14]. IL-17A and IL-17F are the most highly characterized IL-17 cytokines in the context of psoriasis pathogenesis [15]; however, IL-17C is the most abundant IL-17 isoform expressed in lesional human psoriasis skin [16], and is rapidly responsive to efficacious biologics, implicating a potential pathogenic role in disease [15]. To explore this possibility, K5-IL-17C mice were genetically engineered to model increased epidermal IL-17C expression [13], and develop a spontaneous skin phenotype similar to human psoriasis that includes the development of disease after birth (i.e. the phenotype begins to appear at ~6–8 weeks of age), well-demarcated skin lesions with clear gross demarcation between uninvolved and involved skin, an IL-12/23-Th1/Th17 immune cell phenotype, and improvement of skin pathology upon treatment with TNF-α antagonists [13].

This model system offers a unique opportunity to confirm a pro-thrombotic phenotype resulting from chronic skin-specific inflammation and demonstrate the importance of exposure time to inflammation, or chronicity, to this cardiovascular outcome. Our prior work demonstrated keratinocyte-containment of the transgene using genetic-reporter approaches [17]; thus, we hypothesize that K5-IL-17C mice will develop enhanced thrombosis as a direct result of chronic skin-specific inflammation in the presence of elevated circulating proinflammatory monocytes, similar to that observed in the KC-Tie2 mouse model [17]. Of translational importance, recent work done by our group [18] and others (Dr. Nehal Mehta, NHLBI, personal communication) have identified circulating pro-inflammatory monocytes in psoriasis patients that resemble the increased CD11b^+^Ly6C^high^ cells observed in the KC-Tie2 model, and which we hypothesize will also be elevated in K5-IL-17C mice, providing a potential link between chronic skin inflammation and the CVD co-morbidities.

Gene-specific contributions to skin inflammation have recently been investigated pre-clinically using acute (5 days) topical application of Aldara (5 % imiquimod; a TLR7/8 agonist) [19]. Controversy over whether this model is appropriate for studying psoriasis pathogenesis has ensued, in part due to the timing of this elicitation, as well as the lack of chronicity associated with human psoriasis. However, this treatment does model some early events in psoriasiform plaque formation, such as increased acanthosis. Both the K5-IL-17C (chronic) model and the Aldara (acute) model result in increased infiltrating dermal T cells, dendritic cells, and macrophages into lesional tissue [13, 19]. Moreover, similar patterns of elevated pro-inflammatory gene transcripts are observed in lesional skin of both models, including IL-12/23, TNF-α, IL-17A, and IL-17C [13, 19, 20]. These unique experimental models provide the opportunity to compare acute vs. chronic skin-specific inflammation and their effects on systemic monocytosis and thrombosis outcomes.

## Methods

### Mice

K5-IL-17C mice on a C57Bl/6 background were bred and genotyped as previously described [13] in the Case Western Reserve University (CWRU) animal vivarium. Littermates carrying a single non-expressing transgene (either K5tTA or Tet^os^IL-17C) or no transgenes [herein called C57Bl/6, wildtype (WT)] were used as littermate controls. For Aldara experiments, C57Bl/6 mice were purchased (Jackson Laboratories, Bar Harbor, ME, USA) and allowed to acclimatize to the CWRU animal vivarium for at least 14 days before beginning topical application of either Aldara or control cream. All mice used in the experiments were of similar age (10–16 weeks of age), and both male and female mice were used for all experimental outcomes. Average body weights (in grams) were as follows: WT + vehicle (21.8 ± 0.24, n = 21), WT + Aldara (19.8 ± 0.22, n = 25), WT (22.1 ± 1.01, n = 18) and K5-IL-17C (17.3 ± 0.40, n = 14).

For Aldara experiments, mice were shaved 1 day prior to application of either Aldara or control cream (a petroleum-based cream), which was spread on the dorsal surface of the mouse (5 % Aldara, 3 M Pharmaceuticals; 62.5 mg) daily for a period of 5 days. As needed, mice were provided with IP saline to supplement fluid loss associated with Aldara treatment, a well-known side-effect [21]. On day 6, mice underwent the thrombosis protocol outlined below.

Post-hoc statistical analyses examining potential sex-differences were completed. No differences between sexes were observed for any of our reported outcomes thus male and female animals were grouped together for all experimental analyses.

All animal protocols were approved by the CWRU Institutional Animal Care and Use Committee and conformed to the American Association for Accreditation of Laboratory Animal Care guidelines.

### Rose Bengal occlusive thrombosis

The Rose Bengal thrombosis vascular occlusion assay was completed as previously described [17]. Briefly, male and female K5-IL-17C mice [n = 14; male (5), female (9)] or littermate controls [n = 21; male (10), female (11)] or C57Bl/6 WT mice treated with either Aldara [n = 25; male (11), female (14)] or control cream [n = 21; male (10), female (11)] were deeply anesthetized and had their right common carotid artery exposed and monitored by a Doppler flow probe. Animals received a tail-vein injection of Rose Bengal (50 mg/kg) followed by laser illumination of the carotid artery (540 nm) to initiate thrombosis as described previously [22]. Blood flow was monitored until occlusion occurred, defined as cessation of blood flow for 10 min.

### Carotid artery collection post thrombosis

The right common carotid artery of the mouse undergoing the thrombosis procedure was isolated from surrounding tissues prior to thrombosis induction and two silk sutures were placed under the proximal and distal ends of the carotid artery, respectively. A loose knot that did not disturb blood flow was made on each end of the carotid artery. After blood flow was blocked for 10 min continuously as a result of thrombosis induction, the two loose knots were tightened to prevent the leakage of the thrombus clot formed and the artery segment was removed.

### Tissue processing and measurement of carotid diameters

The closed-end-carotid artery with thrombus was then fixed in 4 % formaldehyde (Fisher Scientific, Waltham, MA, USA), and subjected to standard histological paraffin embedding and sectioning. Thin (5 μm) sections with thrombi were collected sequentially and stained with hematoxylin and eosin (H&E). Images were acquired using a Zeiss camera (AxioCam MRc5) linked to a microscope (LEICA DM2000). The diameter of the artery (micrometers) was determined by collecting 4 independent measurements distributed evenly across the cross-section of the measured artery and averaged for each animal.

### Tissue harvesting and flow cytometry

Following thrombosis, skin was harvested and processed as described previously for histology and immunohistochemistry [13, 23]. Skin draining axial and inguinal lymph nodes were isolated from a subset of K5-IL-17C transgenic mice and littermate controls (n = 6 and n = 15; respectively) and Aldara- or control cream-treated C57Bl/6 WT mice (n = 12 and n = 11; respectively) and were then pooled. Spleens were isolated from a subset of K5-IL-17C transgenic mice and littermate controls and Aldara- or control cream-treated C57Bl/6 WT mice (n = 4–9). These tissues were homogenized in serum-free media containing 50 μg/ml DNase I (Sigma, St. Louis, MO) and 2 mg/ml collagenase D (Roche, Basil). Isolated cells were then pelleted, re-suspended, and filtered 2× through a 70 μm filter in wash buffer containing 5 % FBS. The cells were immediately stained for the cell surface markers Ly6C (Alexa Fluor 700; eBiosciences, San Diego, CA, USA), Ly6G (APC; Abcam, Cambridge, MA, USA), and CD11b (eFlour450; BD, Franklin Lakes, NJ, USA). Flow cytometry data collection was performed using a BD FACS-Aria instrument and analyzed using FlowJo software (Tree Star, Ashland, OR, USA).

See Additional file 1: Figure S1 for gating strategy. For monocyte cell gating, monocytes were first selected on an FSC-A vs. SSC-A plot (S1, panel A). The monocytes were then analyzed for live/dead cell populations using the exclusion dye 7-AAD (S1, panel B). From the live monocyte gate, singlet cells were selected and the doublet events were excluded (S1, panel C). Live singlet cells were then selected for CD11b^+^Ly6G^neg^ cells (bottom gate in S1, panel D). From this population, Ly6C was plotted versus SSC-A, and cells that are high on Ly6C and low on SSC-A were considered CD11b^+^Ly6C^high^, excluding CD11b^+^Ly6C^low^ cells and eosinophils (red box in S1, panel E). CD11b^+^Ly6G^+^ neutrophils were gated as shown in panels S1 A, B, and C and were defined as the population in the top gate with dashed black line in S1, panel D.

### Clotting assays

#### Activated partial thromboplastin time

The activated partial thromboplastin time (aPTT) was performed as described previously [24] with modification. Briefly, 50 μl of aPTT reagent (aPTT-SA, Helena Laboratories, Beaumont, Texas, USA) was incubated with 50 μl sodium citrate-anticoagulated plasma at 37 °C for 5 min. Fifty microliters of 30 mM calcium chloride was then added and the time to clot formation was recorded. All samples were tested in duplicate.

#### Prothrombin time

The prothrombin time (PT) was performed as described previously [25] with modification. All required reagents were warmed to 37 ^°^C. Fifty microliters of sodium citrate-anticoagulated plasma was then added into 100 μl of PT reagent (Thromboplastin-LI, Helena Laboratories, Beaumont, Texas, USA) at 37 ^°^C, and the timer was started immediately to record the time to clot formation. Samples were tested in duplicate.

### Statistics

Data analysis and graphs were generated using GraphPad Prism 6 and Microsoft Excel. Results are expressed as mean (±standard error of the mean). Groups were compared using the non-parametric Kruskal–Wallis test. Significance was defined as p < 0.05.

## Results and discussion

**Fig. 1:**
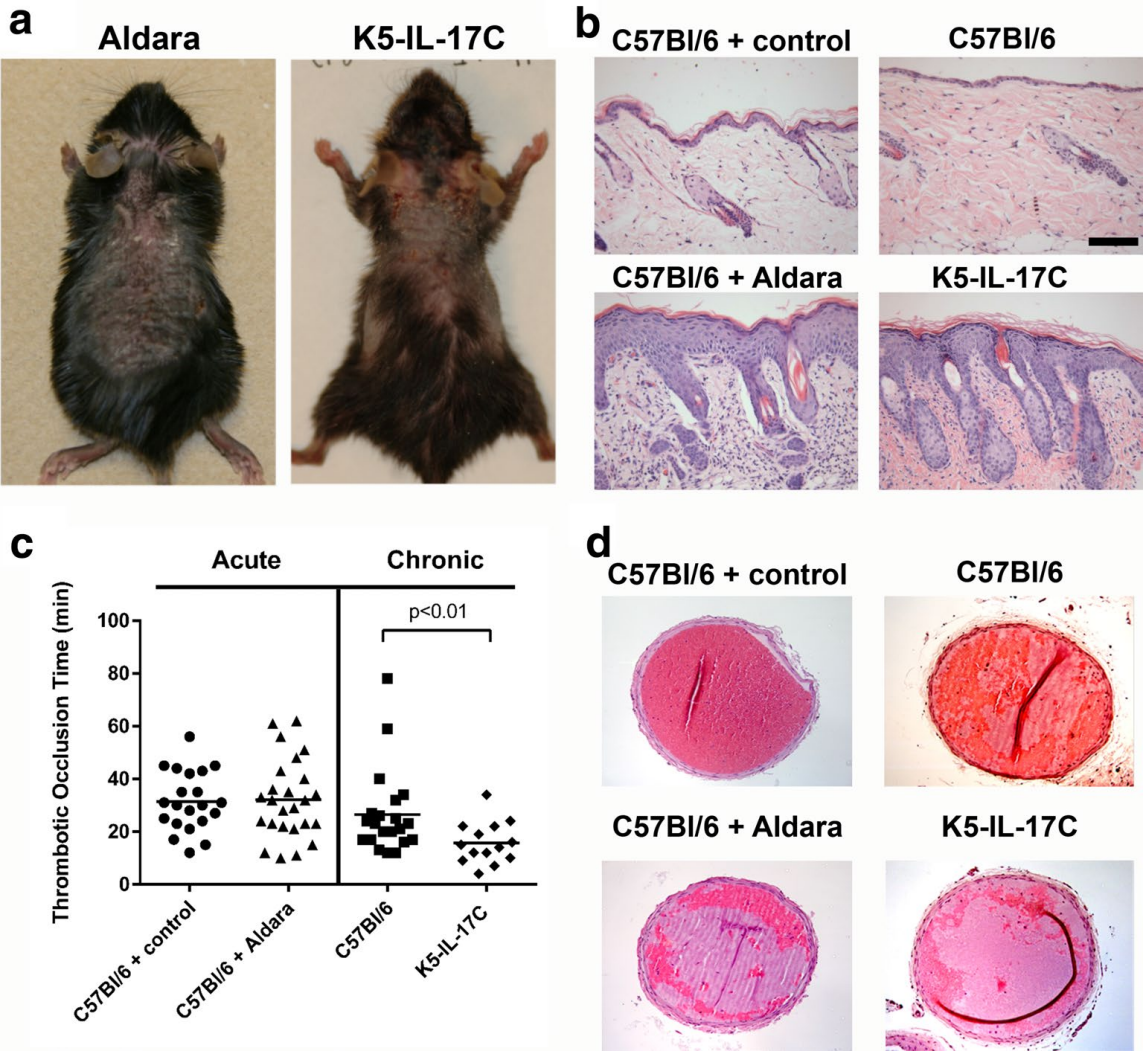
Chronic, but not acute, skin inflammation promotes shorter arterial clotting times in the Rose Bengal thrombosis assay. **a** Representative photographs of a C57Bl/6 WT mouse treated for 5 days with topical Aldara (*left panel*) and a K5-IL-17C mouse (*right panel*). **b** Representative images of H&E stained mouse back skin from a C57Bl/6 WT mouse treated for 5 days with topical Aldara and a K5-IL-17C mouse demonstrate increases in acanthosis compared to controls. **c** C57Bl/6 WT + Aldara clotting times (n = 25) are not different than C57Bl/6 WT + control cream (n = 21); however K5-IL-17C mice (n = 14) have significantly decreased clotting times compared to controls (n = 21), p < 0.01. **d** Representative photographs of cross sections of carotid arteries post-thrombus formation demonstrate no difference in diameter between the groups. *Scale bar* in **b** = 100 μm

Aldara was applied to C57Bl/6 WT mice in an area that approximated the surface area of involved dorsal skin on the K5-IL-17C mice (Fig. 1a) and involved skin from both models developed similar increases in acanthosis (epidermal thickness), an often-used surrogate measure of inflammation for murine skin, compared to control cream-treated and littermate controls (Fig. 1b, bottom row compared to top row). We and others have previously reported the increased presence of skin-infiltrating T cells, myeloid cells, and concomitant increases in pro-inflammatory cell-derived cytokines, including elevated TNF-α, IL-12, IL-23, IL-17A and IL-17C in post-Aldara treated animals and in the chronic K5-IL-17C psoriasiform mouse model [13, 19, 20]. Thus, Aldara, or chronic stimulation of keratinocytes by increased levels of IL-17C leads to cutaneous infiltration of pro-inflammatory cells.

To determine whether the chronicity of skin-specific inflammation affects thrombotic clotting times, we performed the Rose Bengal photochemical carotid artery injury model on the acute inflammatory Aldara-induced model (and control cream-treated mice) as well as the chronic K5-IL-17C mice (and their littermate controls). C57Bl/6 WT mice treated topically with Aldara for 5 days had clotting times similar to C57Bl/6 WT mice treated with control cream (Fig. 1c, triangles vs. circles; 32.2 ± 3.0 vs. 31.4 ± 2.5 min, p = 0.97; n = 25, n = 21, respectively). In contrast, K5-IL-17C mice had significantly reduced times to form occlusive thrombi (enhanced thrombosis) compared to littermate controls (Fig. 1c, diamonds vs. squares; 15.7 ± 2.1 vs. 26.5 ± 3.4 min, p < 0.01; n = 14, n = 21, respectively).

To examine potential differences in carotid arteries post-thrombosis, we harvested, sectioned and stained with H&E, the right carotid artery with the thrombus, from representative mice in each experimental group (Fig. 1d). Diameter measurements of carotids did not differ significantly compared to controls in either the acute Aldara model (385 ± 17.2, Aldara-treated vs. 401 ± 18.1, control cream-treated; n = 9, n = 8, respectively) or the chronic IL-17C model (422 ± 23.5, K5-IL-17C vs. 404 ± 30.6, littermate controls; n = 4, n = 7, respectively) and despite K5-IL-17C mice having the largest carotid artery diameters, they also exhibited the shortest time to occlusive thrombus formation.

To further examine why chronic, and not acute skin inflammation promoted thrombosis, we examined plasma from representative mice to examine ex vivo clotting times and lipid levels. No differences in prothrombin time (PT), an outcome measure that provides insight into influences on the extrinsic coagulation cascade pathway, were observed between Aldara-treated (17.4 ± 1.18 s; n = 7) and control cream-treated mice (15.6 ± 0.94 s; n = 6) and between K5-IL-17C mice (12.8 ± 0.69 s; n = 6) and their littermate controls (13.8 ± 0.17 s; n = 6). We also examined effects of both acute and chronic skin inflammation on the intrinsic pathway of the coagulation cascade, and measured activated partial thromboplastic time (aPTT). No differences were found between experimental groups or model systems (data not shown). Similarly, examination of plasma lipid levels also failed to demonstrate hyperlipidemia in either model system (data not shown).

These data demonstrate that chronic, but not acute, skin inflammation is associated with faster thrombus formation following activation using the Rose Bengal photochemical injury model of thrombosis and validate our prior observations describing shortened thrombosis times in the KC-Tie2-psoriasiform model in a second skin-specific chronic inflammatory mouse model [17]. Others have recently reported increased endothelial dysfunction following chronic skin-specific overexpression of IL-17A (K14-IL-17A), including increased systolic blood pressure, left ventricular hypertrophy, and reduced survival compared with control animals [26]. Collectively, these findings suggest that chronicity and duration of skin-specific inflammation has the capacity to influence the systemic circulation, distant blood vessels, and promote thrombosis.

These preclinical observations are consistent with clinical reports demonstrating that, (1) young psoriasis patients (with moderate-to-severe disease), develop chronic unrelenting systemic inflammation over a sustained period of time and are at highest risk of developing CVD [27]; and (2) psoriasis patients with severe disease, and therefore more significant and prolonged systemic inflammation, have increased risk of thromboembolism [28]. Interestingly, recent epidemiological data suggests that chronicity, or the length of time exposed to persistent inflammation, may play a role in longitudinal increased risk of a myocardial infarction (MI) event, with the more severe psoriasis patients at the greatest risk [29]. Psoriasis patients also had elevated levels of HsCRP (high-sensitivity C-reactive protein), a frequently used measure of systemic inflammation and a surrogate predictor of CVD events [30]. Taken together, these results provide additional evidence that the length of time an individual is exposed to systemic inflammation, such as that derived from inflamed psoriasis skin, appears to increase the risk of developing CVD.

Increased risk of cardiovascular complications has been reported for patients with other chronic organ-specific inflammatory diseases, including inflammatory bowel disease and rheumatoid arthritis, where risk of thromboembolism or levels of unstable carotid artery plaque are also significantly elevated (respectively) compared to healthy controls; during a flare in either disease, this risk further increases [31, 32], and then decreases during disease remission [32]. Whether similar increases occur in psoriasis patients during acute flare remains to be examined.

Inflammation and thrombosis are intertwined in vascular pathology. Observations from pre-clinical and clinical studies indicate that inflammation can beget local thrombosis, and thrombosis can amplify inflammation ([33] and references therein). For example, inflammatory mediators upregulate macrophage tissue factor expression within atherosclerotic plaques, and at the same time, platelets as mediators of thrombosis are critical for the recruitment of inflammatory cells to the vessel wall. Thus, accumulating data linking inflammation and thrombosis support the hypothesis that anti-inflammatory therapies may limit thrombosis and that antithrombotic therapies may reduce vascular inflammation. It is in this context that one must view the contribution of skin-specific inflammation to future adverse cardiovascular events.

Importantly, retrospective meta-analyses suggest some decrease in the incidence of myocardial infarctions in psoriasis patients treated with either TNF inhibitors or anti-IL-12/23 antibody, suggesting that aggressively targeting chronic inflammation and its cellular drivers may reduce life-threatening co-morbidities associated with psoriasis [33, 34]. Our prior findings using the KC-Tie2 mouse model support this, such that reversal of the skin disease, following gene repression, returned thrombosis times to control mouse levels; and reversed aortic root inflammation [17]. Similar improvements in vascular outcomes, including reduced oxidative stress in the heart and blood and attenuation of endothelial dysfunction were reported following TNF-α and IL-6 neutralization in the K14-IL-17A transgenic model [26]. Support for these preclinical findings are now being observed in prospective studies, such that psoriasis patients treated with systemic biologic anti-inflammatory agents show a lower association with CVD events (cardiovascular death, myocardial infarction, stroke) compared to patients treated with other anti-psoriatic therapies [35].

While there was a significant difference in time to occlusive thrombus formation between the acute and chronic models, we observed no difference in PT, aPTT, or lipid profiles, indicating a potential role for an immune cell mediator in the chronic disease model. Previously, we demonstrated that splenic and SDLN directly correlate with, and reflect, circulating pro-inflammatory CD11b^+^Ly6C^high^ monocytes in the chronic, skin-specific KC-Tie2 model [17]. Furthermore, we reported that these cells are increased in KC-Tie2 mice in the presence of enhanced thrombosis [17] and we recently validated the elevated presence of the human homolog (CD14^+^CD16^++^ intermediate monocytes) in psoriasis patient blood [18]. Finally, increased levels of circulating monocytes have been previously categorized as a risk factor for coronary heart disease [36], indicating that monocytes may participate in the cardiovascular outcomes of the chronic disease model.

**Fig. 2:**
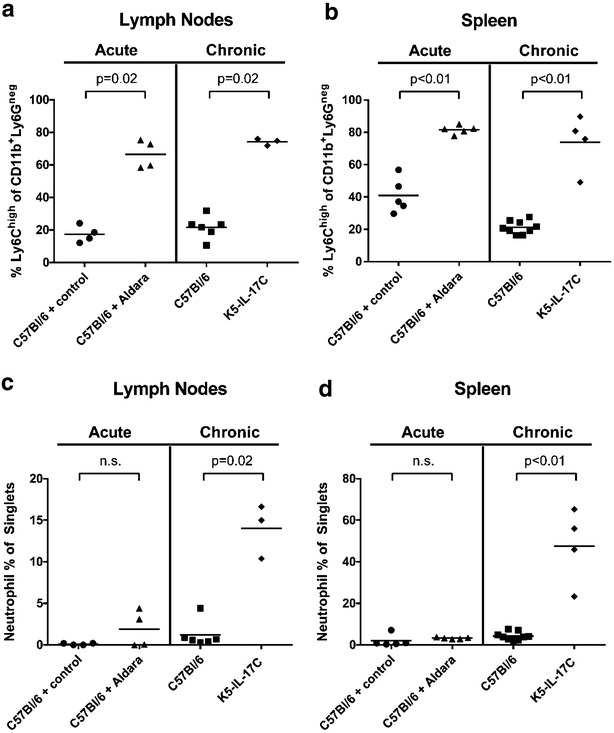
CD11b^+^Ly6C^high^ monocytes increase in both Aldara-treated and K5-IL-17C mice whereas CD11b^+^Ly6G^+^ neutrophils increase only in K5-IL-17C mice. **a** Representative flow cytometry dot plots of CD11b and Ly6C surface staining in skin-draining axillary and inguinal lymph nodes (LN). C57Bl/6 WT mice treated for 5 days with topical Aldara (n = 4 pooled samples) and K5-IL-17C mice (n = 3 pooled samples) have increases in skin-draining LN-CD11b^+^Ly6C^high^ cells compared to their respective controls (n = 4 and n = 6 pooled samples; p = 0.02 and p = 0.02, respectively). Each point represents lymph nodes pooled from 2 to 3 animals. **b** Increased splenic-CD11b^+^Ly6C^high^ are also observed in Aldara-treated WT mice (n = 5) and K5-IL-17C mice (n = 4) when compared to their respective controls (n = 5, n = 9; p < 0.01 and p < 0.01, respectively). Each point represents a single animal. **c** Aldara-treated mice have similar levels of SDLN-derived neutrophils (CD11b^+^Ly6G^+^) as control-cream treated mice (n = 4, n = 4) whereas SDLN CD11b^+^Ly6G^+^ cells are significantly increased in K5-IL-17C mice compared to littermate controls (n = 3 pooled samples, n = 6 pooled samples; p = 0.02, respectively). **d** Aldara-treated mice have similar levels of splenic CD11b^+^Ly6G^+^ cells as control-cream treated mice (n = 5, n = 5) and K5-IL-17C mice have significant increases in splenic CD11b^+^Ly6G^+^ cells compared to littermate controls (n = 4, n = 9 pooled samples; p < 0.01, respectively)

To determine if the observed increase in frequency of pro-inflammatory CD11b^+^Ly6C^high^ monocytes may contribute to the promotion of thrombosis, we isolated skin-draining axial and inguinal lymph node (SDLN) cells from a subset of acute Aldara-treated C57Bl/6 WT mice, chronic K5-IL-17C animals, and their relative controls. Using flow cytometry, we measured CD11b, Ly6G, and Ly6C on the surface of SDLN cells (representative image in Additional file 1: Figure S1E, red box). Interestingly, CD11b^+^Ly6C^high^ pro-inflammatory monocytes were significantly increased in both the acute (Aldara-treated) and chronic (K5-IL-17C) skin inflammation models compared to their respective controls in both the SDLN (Fig. 2a; 66.6 ± 4.4, Aldara-treated vs. 17.4 ± 2.6, control-cream treated; p = 0.02, n = 4, n = 4; and 74.2 ± 1.2, K5-IL-17C vs. 21.6 ± 2.8, littermate controls; p = 0.02, n = 3, n = 6; respectively) and spleen (Fig. 2b; 81.7 ± 1.2, Aldara-treated vs. 40.9 ± 4.8, control-cream treated; p < 0.01, n = 5, n = 5; and 73.9 ± 8.7, K5-IL-17C vs. 21.2 ± 1.3 littermate controls; p < 0.01, n = 4, n = 9; respectively). These data suggest that CD11b^+^Ly6C^high^ monocytes accumulate rapidly in draining SDLN and spleen following skin inflammation in both the acute and chronic models. Despite the increase in spleen- and SDLN-CD11b^+^Ly6C^high^ cells, time to occlusive thrombosis formation failed to change significantly between Aldara-treated and their respective control mice (Fig. 1c), suggesting that acute monocytosis alone is not prothrombotic.

In other chronic illnesses, such as HIV, increased levels of circulating lymphocytes and leukocytes have also been observed and proposed to be responsible for the increased risk of cardiovascular events [37]. However, more important than numerical increases, perhaps, is the functional activation of these monocytes and lymphocytes, as suggested by Funderberg et al. [38], who demonstrate that monocytes can become activated by exposure to oxidized LDL (oxLDL), leading to increased cardiovascular risk. Interestingly, psoriasis patients are dyslipidemic and have increased circulating and plaque oxLDL [39]. Moreover, stimulation of macrophages with psoriasis patient-isolated LDL increases production of IL-6 and TNF-α, and also results in increased monocyte adhesion to human umbilical vein endothelial cells [40]. Thus, increases in oxLDL found in psoriasis patients and oxLDL-mediated effects on lymphocytes may provide further support for how skin-inflammation promotes distant vessel inflammation and atherothrombosis. In the acute Aldara and chronic K5-IL-17C model systems, mice did not develop hyperlipidemia (data not shown), consistent with prior observations in KC-Tie2 mice [17], suggesting that the promotion of thrombosis occurs independent of lipid-mediated effects on lymphocytes in these model systems.

Other chronic inflammatory diseases have associated increased cardiovascular risk, and chronic inflammation can extend to inflamed vessels (arteries) that are likely to signal for additional pro-inflammatory leukocytes and lymphocytes [41], leading to increased infiltration of pro-inflammatory cells. This contextual activation of both monocytic and endothelial cells may be dependent on the length of exposure to chronic inflammation; such that increased exposure, like that induced in the chronic K5-IL-17C model, but not acute exposure, similar to that elicited by Aldara application, are necessary to compromise endothelial-monocyte (dys)function. Supporting this concept are reports that recent-onset plaque psoriasis patients fail to present with endothelial dysfunction [42] whereas established-plaque psoriasis patients do [43], consistent with the idea that disease duration may lead to the observed dysfunction in chronic patients. Additional observations in an additional preclinical model of psoriasiform skin involvement also demonstrate that overexpression of IL-17A leads to systemic endothelial dysfunction, suggesting that chronic skin inflammation may result in vascular changes indicative of predisposition to eventual cardiovascular dysfunction.

Along with endothelial dysfunction, psoriasis patients also have increases in circulating endothelial cells and microparticles (MPs), which may promote coronary artery disease, acute coronary syndromes and atherothrombosis [44, 45]. Importantly, these decrease following treatment with anti-TNF-α therapy [44] and could provide mechanistic insight into why TNF-α inhibition reduces risk of MI in psoriasis patients [46].

In addition to MPs and endothelial cells, other cellular mediators of thrombosis such as neutrophils have been previously reported to be elevated in preclinical psoriasis models [26] and human psoriasis patients [47]. Therefore, we also examined CD11b^+^Ly6G^+^ neutrophils in both spleens and lymph nodes of the chronic and acute psoriasiform models. In the acute Aldara-treated mice, no increases in either SDLN or splenic resident neutrophils were observed compared to control-cream treated mice (Fig. 2c; SDLN: 1.90 ± 1.10, Aldara-treated vs. 0.10 ± 0.06, littermate controls, p = 0.37, n = 4, n = 4; respectively; Fig. 2d. Spleen: 3.26 ± 0.17, Aldara-treated vs. 2.25 ± 1.62, littermate controls; p = 0.28, n = 5, n = 4; respectively). In contrast, K5-IL-17C mice, had elevated neutrophil percentages when compared to littermate controls in both the SDLN (Fig. 2c; 14.0 ± 1.87, K5-IL-17C vs. 1.21 ± 0.65, littermate controls, p = 0.02, n = 9, n = 4; respectively) and spleen (Fig. 2d; 47.6 ± 9.0, K5-IL-17C vs. 4.23 ± 0.66, littermate controls; p < 0.01, n = 9, n = 4; respectively).

Elevated monocytes and neutrophils, the activation status of these recruited immune cells, or potentially the micro-milieu the monocytes and neutrophils encounter, may all contribute to the thrombotic potential of the mice. Depletion of neutrophils from an alternative chronic skin inflammation model [26] resulted in decreased reactive oxygen species in peripheral blood, although changes in endothelial dysfunction were not examined. Targeted experiments designed to eliminate monocyte egress from the bone marrow, thus depleting these cells from circulation in psoriasiform mice (i.e., backcross CCR2^−/−^ and/or CCR5^−/−^ mice with either KC-Tie2 or KC-IL-17C) should address the necessity and importance of monocytosis for thrombosis alterations. In addition, alternative cellular mediators of inflammation, such as neutrophil extracellular traps (NETs) [48] may play additional, as yet unidentified roles, in the thrombotic process as suggested previously by other investigators, using the Rose Bengal model of thrombosis [49, 50]. Duration, or chronicity of the skin inflammation, appears to be more correlative with shortened thrombosis times rather than accumulation of pro-inflammatory cellular mediators. However, the duration of the cutaneous cellular response and resultant prolonged exposure to immune cells and derived cytokines may also be a critical factor rather than the appearance of transient pro-inflammatory cells.

## Conclusions

Acute, as well as chronic, skin-specific inflammation promotes the circulation and infiltration of proinflammatory CD11b^+^Ly6C^high^ monocytes into the skin, however, significant changes in neutrophil percentages and occlusive distant vessel thrombosis (following induction by Rose Bengal) occurs only in animals with chronic skin-specific inflammation. Our results provide evidence in an independent second genetic skin-contained mouse model that chronic cutaneous inflammation promotes faster thrombosis following Rose Bengal photoinjury; however our findings also suggest that despite similar levels of skin involvement (body surface area) in acute and chronic models, the length of exposure to skin-elicited inflammation, and elevated cellular participants, such as neutrophils, appear critical to pathogenic outcomes. Further work delineating the cellular and molecular response to psoriasis that promotes inflammation and poor CVD outcomes, at the pre-clinical and clinical levels, are needed to better understand the link between psoriasis and CVD.

## Additional file

10.1186/s12967-015-0738-z Gating strategy to identify the CD11b^+^Ly6G^neg^Ly6C^high^ monocyte population and CD11b^+^Ly6G^+^ neutrophils. (A) FSC-A vs. SSC-A plot was used on all events to identify monocytes. (B) The monocytes were next analyzed for live/dead cell populations based on 7-AAD cell exclusion. (C) From the live monocyte gate, cells were gated for singlet and doublet events, and the doublet events were excluded. (D) The singlet cells were selected for CD11b^+^Ly6G^neg^ cells (based on istoypes for CD11b and Ly6G). The black, dashed line indicates the gate used to collect CD11b^+^Ly6G^+^ neutrophils. (E) From CD11b^+^Ly6G^neg^ gate, Ly6C expression was plotted versus SSC-A, and cells that expressed high levels of Ly6C (based upon isotype) and low on SSC-A (i.e., non-granular) were considered CD11b^+^Ly6C^high^ monocytes. Cells that expressed low levels of Ly6C (based upon isotype) and low on SSC-A (i.e., non-granular) and eosinophils (high on SSC-A) were excluded.

## Abbreviations

aPTT: activated partial thromboplastin time
CVD: cardiovascular disease
hsCRP: high-sensitivity C-Reactive Protein
IL-17C: Interleukin-17C
SDLN: skin-draining lymph node
MI: myocardial infarction
MP: microparticle
NET: neutrophil extracellular trap
oxLDL: oxidized LDL
PT: prothrombin time
TNF-α: Tumor necrosis factor alpha
WT: wild type
