# Supplementary figures and images for: Chronic, not acute, skin-specific inflammation promotes thrombosis in psoriasis murine models
Source: J Transl Med. 2015 Dec 16;13:382. doi: 10.1186/s12967-015-0738-z (PMC4681031; doi:10.1186/s12967-015-0738-z)

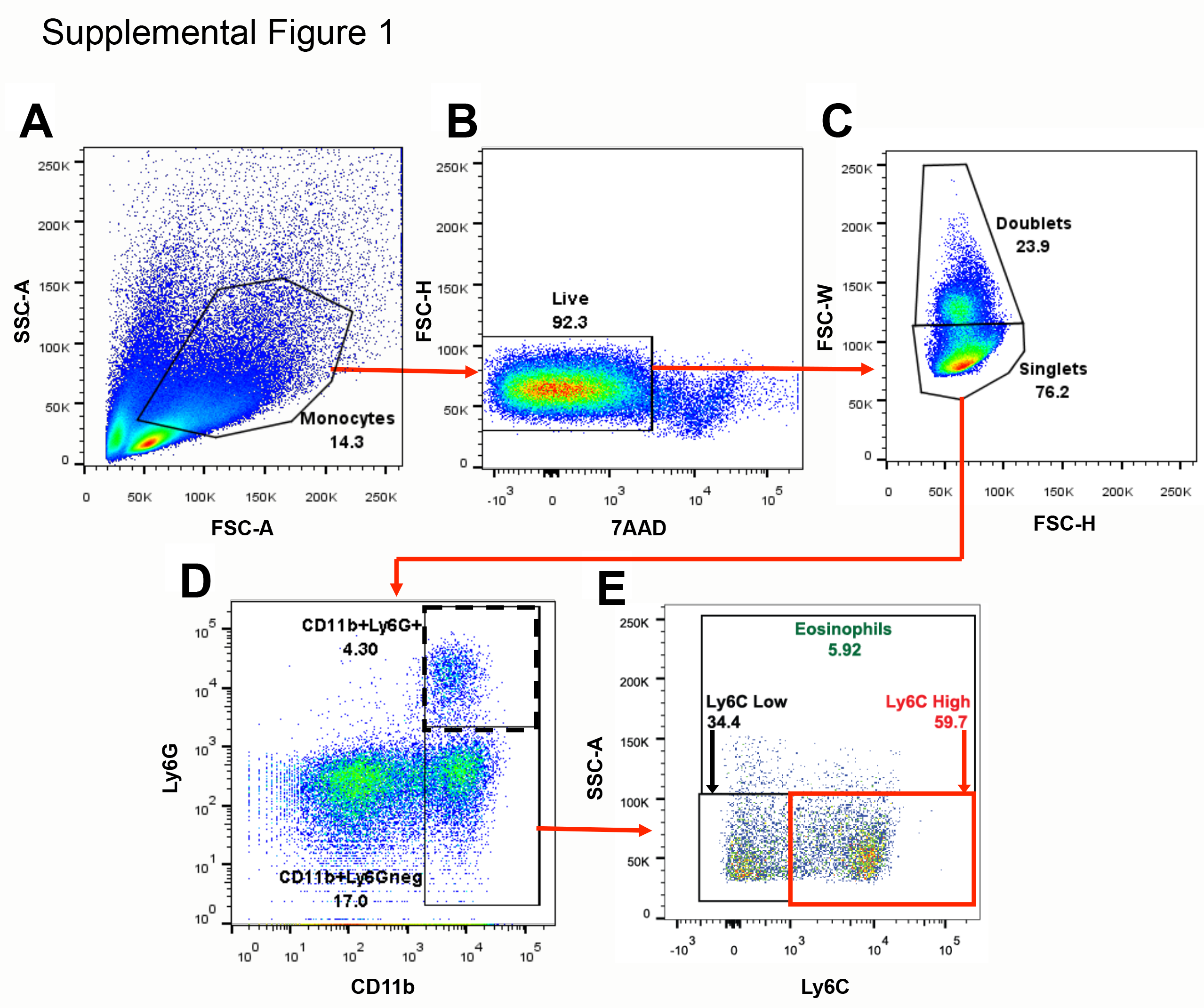

Supplement: Supplementary file 1 — 10.1186/s12967-015-0738-z Gating strategy to identify the CD11b+Ly6GnegLy6Chigh monocyte population and CD11b+Ly6G+ neutrophils. (A) FSC-A vs. SSC-A plot was used on all events to identify monocytes. (B) The monocytes were next analyzed for live/dead cell populations based on 7-AAD cell exclusion. (C) From the live monocyte gate, cells were gated for singlet and doublet events, and the doublet events were excluded. (D) The singlet cells were selected for CD11b+Ly6Gneg cells (based on istoypes for CD11b and Ly6G). The black, dashed line indicates the gate used to collect CD11b+Ly6G+ neutrophils. (E) From CD11b+Ly6Gneg gate, Ly6C expression was plotted versus SSC-A, and cells that expressed high levels of Ly6C (based upon isotype) and low on SSC-A (i.e., non-granular) were considered CD11b+Ly6Chigh monocytes. Cells that expressed low levels of Ly6C (based upon isotype) and low on SSC-A (i.e., non-granular) and eosinophils (high on SSC-A) were excluded. [file 12967_2015_738_MOESM1_ESM.tiff]
